# Thermal-Oxidative Aging Effects on the Dielectric Properties of Nuclear Cable Insulation

**DOI:** 10.3390/ma13102215

**Published:** 2020-05-12

**Authors:** Xiaohong Chi, Jianxi Li, Minzun Ji, Wenfeng Liu, Shengtao Li

**Affiliations:** 1 State Key Laboratory of Electrical Insulation and Power Equipment, Xi’an Jiaotong University, Xi’an 710049, China; hrbustcxh@xjtu.edu.cn (X.C.); jiminzun@stu.xjtu.edu.cn (M.J.); sli@mail.xjtu.edu.cn (S.L.); 2 CGN-DELTA Testing Technology CO., LTD., Taicang 215400, China; lijianxi@cgnam.cn

**Keywords:** nuclear power, cable insulation, thermal-oxidative aging, dielectric properties

## Abstract

In order to satisfy demands of cable insulation for nuclear power, a low-smoke, halogen-free flame retardant and better cryogenic property insulation was prepared. The effect of thermal-oxidative aging on the dielectric properties was researched in this paper. The changes of chemical structure and thermal-oxidative stability caused by aging were characterized by Fourier transform infrared spectroscopy and a differential scanning calorimeter. The results showed that, the oxidative-induced stability reduced as complex products accumulated during aging. The dielectric properties including polarity, conductivity and breakdown at different aging stages were measured. For comparison, tensile testing was performed. The parameters related to conductivity changed notably, and were comparable with the changes in mechanical properties.

## 1. Introduction

Nuclear power energy, with the characteristics of safety, cleanliness and efficiency, has been developing for more than 60 years. Worldwide, there are 450 nuclear power reactors in operation, totaling 396 GW(e) of generation capacity. According to the information published by the International Atomic Energy Agency (IAEA), in 2018, total nuclear power generation was 2563 TWh. The proportion of nuclear power in global power generation was about 10.5%. The proportion of nuclear power in national electricity generation was more than 10% in 20 countries, more than 25% in 30 countries and more than 50% in 4 countries [1,2].

Nuclear power has developed fast in China. As of the end of June 2019, the installed capacity of commercial nuclear power was 36,933.16 MWe (rated installed capacity). From January to March, 2019 in China the capacity of cumulative power generation was 16.75 TWh, while the nuclear power was 0.77 TWh, accounting for about 4.59% of the total. Compared with coal-fired power generation, nuclear power was equivalent to reducing the combustion of 23.66 million tons of coal, and preventing 61.98 million tons of carbon dioxide, 201,100 tons of sulfur dioxide and 175,100 tons of nitrogen oxide emissions [3]. The clean energy industry is growing steadily, and the role of nuclear power in clean energy is prominent. The demand for electricity will continue to grow, and nuclear power still has a large development space. In the future, nuclear power will continue to increase to meet the needs of economic development and environmental protection [4,5,6,7].

The construction and development of nuclear power have put forward higher requirements and demands for the cable industry. The service life of the advanced third-generation nuclear power plants (NPPs) have been increased to 60 years [8]. Because of the particularity of cables in NPPs, many of them cannot be replaced after installation. Therefore, the design of the service life of cables for NPPs should also be more than 60 years. For the control cables and low voltage cables in NPPs, which are less irradiated, thermal-oxidative aging is the critical factor of degradation [9].

In the aging process of cable insulation, the change of mechanical properties is obvious. The elongation at break (EAB) is often used to judge the end of the cable insulation life [10,11]. However, the destructive test of EAB cannot collect data online and in real time. Dielectric properties are important application characteristics of insulation, which directly affect the stable operation of cables [11,12,13]. Significant electrical property variations with aging have already been observed at very low frequencies (down to 10^−6^ Hz) and relatively high frequencies (1–2 GHz). However, it is still necessary to correlate these results with those obtained by mechanical tests. Changes of electrical properties at specific frequencies may be chosen as aging markers for NPP cables, in comparison with the mechanical modifications which can be harmful for cable reliability [14].

At present, in a nuclear power plant, there are many polymer matrix materials for the insulation of medium- and low-voltage cables. However, at low temperature, it is difficult for the existing insulation of cables to maintain performance. To develop insulation for cables with good low-temperature performance, ethylene-vinyl-acetate (EVA) was used as polymer matrix and blended with low-density polyethylene (LDPE) to prepare low smoke and halogen-free flame-retardant insulation materials. After thermal-oxygen aging carried out under the same conditions, the mechanical tensile tests of the new formula and the industrial cable insulation was carried out at 0 °C. With the increase of aging time, the EAB of the two industrial materials decreased significantly. Therefore, the blends of EVA and LDPE meet the application requirements at room temperature and have better low-temperature characteristics [15]. In this research, the new formula of cable insulation for NPPs which had better low temperature performance compared with the existing insulating materials was prepared. After the thermal-oxidative aging treatment, we tested the changes of properties of cable insulation with different aging time, including the chemical, thermal, dielectric and mechanical properties, so as to grasp the action law of aging on the deterioration of dielectric properties.

## 2. Materials and Methods

### 2.1. Samples Preparation

A new formula of cable insulation for NPPs was prepared. The composites were a compound of ethylene-vinyl-acetate (EVA, E180F, Samsung General Chemicals Co., Ltd., Seosan-shi, KR,0.93g/cm^3^, 18% vinyl-acetate content), polyethylene (LDPE, 7042, SINOPEC QILU Company, Zibo, China, 0.92g/cm^3^), magnesium hydroxide (Mg(OH)_2_, Albemarle Corporation, America, AR, 95%), antioxidant (KY45, Xinchang Chemical Co., Ltd., Nantong, China, nano-montmorillonite (MMT), and other supplements such as crosslinking agents, voltage stabilizers and processing aids, etc (all chemicals was prepared by CGN-DELTA Testing Technology). The weight fraction of each component in the formula was as follows: EVA was 38 wt%; PE was 2 wt%; Mg(OH)_2_ was 48 wt%; MMT was 2 wt%; antioxidant was 2 wt%; other supplements were 8wt%. Blend the ingredients evenly at the processing temperature that the polymer was full melting. After blending, the plate samples were prepared by hot pressing and the crosslinking process was completed by 120 kGy radiation (Co60, 7.8 kGy/h). Samples were cut into different sizes according to test requirements.

### 2.2. Thermal-Oxidative Aging

The artificial accelerated aging was carried out in an air-circulating oven. According to IEC60216-2001 standard, the thermal-oxidative aging temperature was 165 °C. The tests of mechanical, dielectric, thermal and chemical properties were performed on samples at aging time of 0 h, 48 h, 96 h, 168 h, 336 h, 504 h, 672 h and 840 h.

### 2.3. Chemical and Thermal Testing

The chemical structural changes during aging were analyzed with a Fourier transform infrared (FTIR) spectrometer (Nicolet iN 10MX, Thermo Scientific, Waltham, MA USA). The change of the polymer chains’ arrangement in the sample during ageing could be shown in the process of melting and the oxidative-induced reaction. In order to characterize the effect of aging on the melting and oxidative-induced action of samples, differential scanning calorimeter (DSC) tests were carried out at different aging stages (DSC 882e, Mettler-Toledo Inc., Greifensee, Switzerland). The sample weight of about 10 mg was used for dynamic oxidative induction. To remove the air, nitrogen was injected into the chamber for 5 min before heating, and then heated in pure oxygen at the heating rate of 10 °C/min. The staring of decomposition temperature of EVA is about 350 °C, so the temperature range of DSC measurements was from 50 °C to 350 °C.

### 2.4. Dielectric Properties Testing

Recommended standard of IEC60093, the DC volumetric resistivity was measured with a electrometer (6517A electrometer, Keithley, Cleveland, OH, USA) and the test was performed with a system of three-terminal electrodes at room temperature. The loss current result was recorded after applying a DC voltage of 500 V for 1 min, and the volumetric resistivity was calculated by the loss current and thickness of specimen.

The AC breakdown experiment was conducted by a computer-controlled voltage breakdown tester (HJC-100KV, Huabotech Co., Ltd., Changchun, China). According to standard of IEC60243-1, a sphere-sphere copper electrode with 25 mm diameter was used in breakdown tests. The thickness of samples for breakdown was about 1 mm. The electrode and sample were immersed in the insulation oil to avoid surface flash when applying voltage. The frequency of alternating current (AC) voltage was 50 Hz and the rate of rising voltage was 2 kV/s. The voltage values were recorded at breakdown and the breakdown strength was calculated by the breakdown voltage and thickness of samples. At least 30 breakdown tests were conducted on the samples in each aging stage, and the average value and standard error (SE) were calculated.

The frequency dependency of permittivity and loss were measured with broadband dielectric spectrometer (Concept 80, Novocontrol, Montabaur, Germany). Gold electrodes with a diameter of 30 mm were sputtered on the sample film, and the test was performed at 60 °C with the frequency ranging from 0.1 Hz to 1 MHz.

### 2.5. Tensile Testing

According to the experimental standard of ISO 527-3, the tensile testing was carried out by an electronic universal testing machine (CMT4503, SANS, Shenzhen, China). The experimental sample was cut into a 4 mm × 1 mm dumbbell with a measurement length of 20 mm, and the tensile rate was 100 mm/min. At least five samples of each group were measured to obtain an average value.

## 3. Results and Discussion

### 3.1. Chemical Structural Analysis

**Figure 1:**
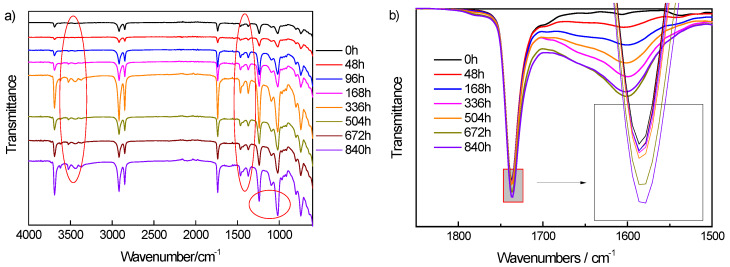
The absorption spectrum of Fourier transform infrared (FTIR) spectroscopy of the samples at different aging stages, **a**) was the absorption spectrum from 4000 to 1500 cm^−1^ and **b**) was the smaller step size scanning in the range of 2000–1500 cm^−1^.

The chemical changes during the aging process were characterized by Fourier transform infrared (FTIR) spectroscopy, and the absorption spectrum of samples at different aging stages were shown in Figure 1. The chains of polyethylene (PE) had strong absorption peaks at 2870 cm^−1^, 1460 cm^−1^ and 720 cm^−1^, respectively; 2870 cm^−1^ corresponded to the stretching vibration of the C–H bond of methylene. As for the symmetrical and asymmetrical stretching vibration, the absorption peak at 2870 cm^−1^ was a splitting peak [16]. Bending vibration absorption of the methylene C–H bond was at 1460 cm^−1^, and rocking vibration absorption of that was at 720 cm^−1^. EVA had the typical absorption peaks of ester, such as the stretching vibration of C=O bond of carbonyl ester at 1736 cm^−1^, C–O–C vibration of ether bond at 1300–1000 cm^−1^, and the other absorption peaks of the C–H bond coincided with PE [17].

The absorption peaks corresponding to aging products increased with the aging time, which indicated that chemical changes had taken place in the molecular chains. Figure 1a shows the absorption spectrum of FTIR. As one of the aging products, the absorption peaks of carboxylic acid were at the wave numbers of 3391 cm^−1^ and 3369 cm^−1^ [18]. The peaks of carboxylic acid were not obvious at the early stage of aging, but increased notably due to the accumulation of carboxylic acid groups after aging for 336 h. The absorption peak at 1020 cm^−1^ was the vibration superposition of C–O bonds in ester and alcohol which was the aging product [19]. With the prolonging of aging time, the absorption peak of 1020 cm^−1^ increased more obviously than that of 1240 cm^−1^, which indicated that the cumulative primary alcohols and carboxyl groups increased continuously during aging process.

There were many C=C bonds in the aging products, but the absorption peak of the C=C bond in the whole spectrum was lower than the intrinsic group. In order to characterize the change of double bonds, a measurement with a smaller scanning step size was carried out in the range of 2000–1500 cm^-1^, as shown in Figure 1b. The peak in 1750–1730 cm^−1^ was the carbonyl absorption. With the aging time prolonged, the absorption intensity and the area of the carbonyl peak increased. This was due to the thermal oxygen reaction of insulating materials under long-term thermal aging. Free radicals caused by the C–C bond rupture were combined with oxygen to form carbonyl groups. At 1600 cm^−1^, the absorption peak of C=C bond increased with aging time. The oxidative cracking reaction which was accelerated during aging resulted in massive aging products containing C=C bond [20].

### 3.2. Melting and Oxidative-Induced Properties

**Figure 2:**
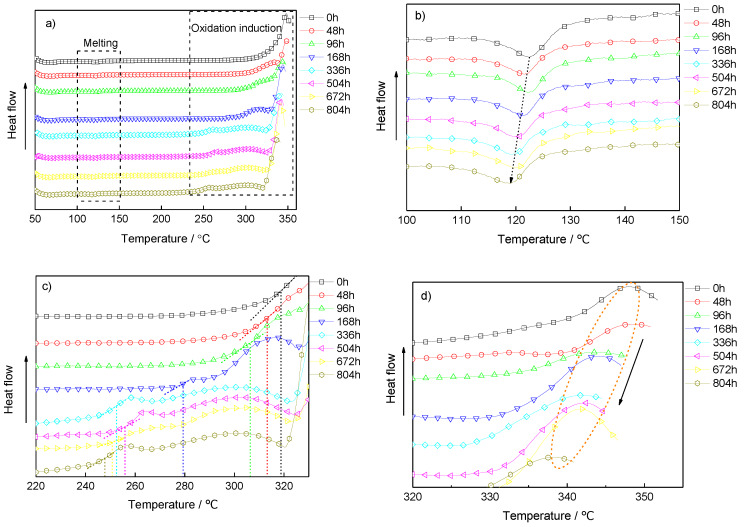
The effects of aging on the melting and oxidative inducted behavior of the samples measured by a differential scanning calorimeter (DSC), (**a**) DSC curve in the whole heating process, (**b**) melting endothermic process, (**c**) the onset of oxidative-induced temperature (OIT), (**d**) the peak of OIT.

The effects of aging on the melting and oxidative-induced behavior of the samples were shown in Figure 2a. In order to characterize the melting and oxidation induction characteristics clearly, the processes were separated. Figure 2b shows the melting endothermic process. Figure 2c,d show the onset and peak of the oxidative-induced temperature (OIT) of polymers. 

Figure 2b shows that there was only one melting peak, although the sample was a blend polymer of PE and EVA, which indicates that the two polymers were completely compatible [21,22]. With the increase of aging time, the melting temperature of the sample decreased continuously. The peak of melting temperature of the unaged sample was about 122 °C, and that of the sample aging for 804 h decreased by nearly 4 °C. This was due to the amount of reactions, such as crosslinking and degradation of molecular chains, which influenced the movement of molecular chains of the polymer, destroyed the original crystalline structure, and formed small grains and incomplete crystals. The new structures melted at a lower temperature.

The OIT of polymers showed the properties of the antioxidant [23]. From Figure 2c,d, it can be seen that the onset and peak of OIT of the samples decreased with the thermal-oxidative aging degree. This was because the massive free radicals caused by aging could react with oxygen at a lower temperature and lead to the exothermic from the chemical bond breaking of polymers. After aging more than 100 h, the reaction period of the samples became longer, and there was an obvious oxidation induction period before obvious thermal degradation occurred. This was because the high active free radicals which was produced during aging could take place reactions at lower temperatures [24].

### 3.3. Dielectric Properties

Polarization, loss, conductivity and breakdown were intrinsic parameters of dielectric materials, which needed to be obtained by different and appropriate test methods. The intrinsic loss of insulating materials includes conductivity loss and relaxation polarization loss, so it is necessary to test the loss of samples at different frequencies. To avoid the influence of space charge polarization, low voltage was applied in the measurement of the intrinsic loss of the polymer at different frequencies. In the measuring the conductance current, the application voltage was the service voltage, and the leakage current of the sample included electronic conductivity and ionic conductivity. In the breakdown testing, the dielectric strength of the sample was measured with the increasing voltage at a certain speed until the sample breakdown. Because of the different parameters concerned in different tests, the voltage used in the dielectric spectrum was different from the conductivity and breakdown tests.

#### 3.3.1. Polarized and Loss Characteristics

**Figure 3:**
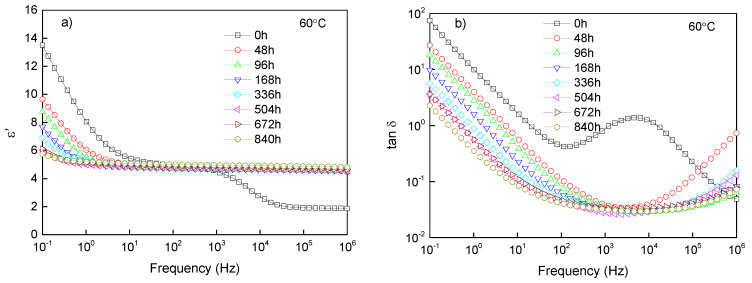
The dependency of real permittivity and loss on frequency at the service temperature of cables, (**a**) the spectrum of real permittivity, and (**b**) the spectrum of tanδ.

Permittivity was a parameter to characterize the polarization characteristics of insulation. The response of polar groups under an electric field can be characterized by the change of permittivity with frequency. In order to obtain the polarization characteristics of cable insulation at different aging stages, the real permittivity ε′ and dielectric loss tanδ of samples were measured at 60 °C, as shown in Figure 3.

In the low-frequency region, it could be seen that the real permittivity and loss of the samples after aging were lower than those without aging. The decrease of real permittivity and loss in low frequency was related to the decrease of polarization and conductance [25]. The arrangement and structure of polymer chains were changed owing to the degradation of molecular chain, then influenced the energy levels. The cross-linking reactions during aging made it difficult for dipoles to polarize under test conditions, which reduced the real permittivity. The introduction of a double bond formed a deep level and restrained the directional migration of carriers under the electric field, resulting in the decrease of the conductive current and the conductive loss at low frequency accordingly.

In the high-frequency region, the real permittivity of the aged samples was higher than that of the unaged sample. The random breakage of molecular chains caused by aging enlarged the distribution of molecular weight, which introduced dipoles with different intensities [26]. Dipoles produced by aging could polarize in a wide frequency range, which showed that the real permittivity and loss of aged samples were higher than those of unaged samples in the high-frequency range. 

#### 3.3.2. Conductivity Characteristic

**Figure 4:**
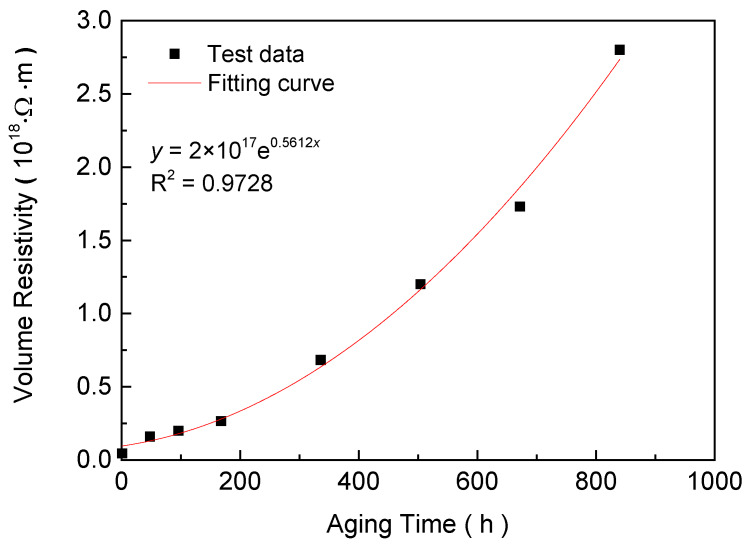
Variation of volumetric resistivity versus the aging time.

Volumetric resistivity was an important parameter for insulation, which could influence its loss and service life. The value of resistivity was obtained by measuring the stable leakage current flowing through the sample under direct current (DC) voltage. Each sample was tested eight times, the average leakage current was obtained, and the volume resistivity was calculated by the average value. Volumetric resistivity of insulation was an intrinsic parameter of material, which was independent of the shape of the sample. It was mainly related to the concentration and mobility of carriers in the sample under applied voltage. Under an electric field, the source and directional motion of carriers in samples were affected by the molecular chain structure and aggregation, which changed with aging time. The variation of volume resistivity of samples at different aging stages is shown in Figure 4. 

The curve fitting of the volume resistivity with the aging time was carried out. When the aging time is 0–804 h, the volume resistivity with the aging time were exponentially related, and the goodness of fit was 0.98. It could be seen that the volumetric resistivity of the sample increased obviously with aging time, which indicated that aging affected the concentration and directional motion of carrier. According to the results of FTIR, oxygen-containing groups and double-bond structures formed during aging introduced deep trap states in the sample [27]. The charge would be trapped and find it difficult to escape [28]. In the test field strength, the carrier in the sample would be trapped instead of migrating to form a steady current, which led to the increase of the resistivity.

#### 3.3.3. Breakdown Strength

**Figure 5:**
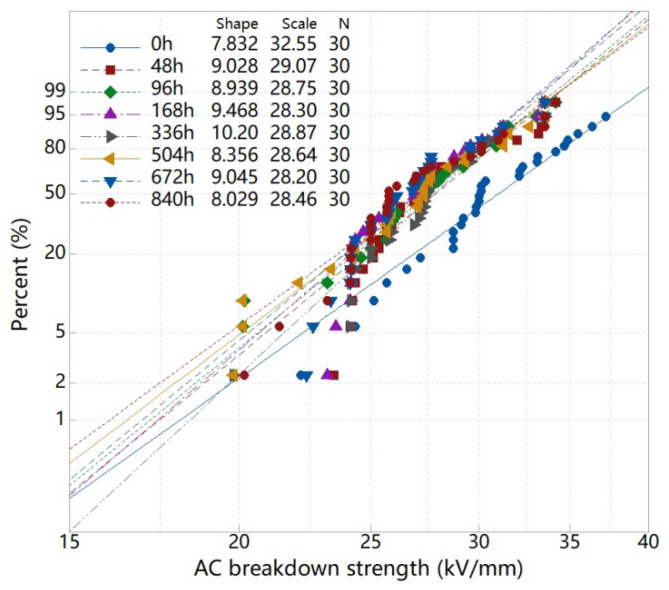
AC breakdown strength of samples with different aging time.

The breakdown strength was an important parameter to evaluate the insulation reliability of the material. In this study, the AC breakdown strength was measured. The failure of dielectrics followed a certain probability, and the Weibull distribution was used to analyze the breakdown data. The scale parameter α, which is known as the characteristic breakdown strength (kV/mm), is the strength at which the breakdown probability was 63.2%. The shape parameter β represented the dispersion of breakdown data. The Weibull distribution of breakdown strength results are shown in Figure 5. It could be seen that the breakdown strength of samples decreased at the beginning of aging, compared with the samples without aging. After that, the breakdown field strength of the sample hardly changed with aging time.

Generally, the breakdown and aging of insulating were closely related to the changes of the internal structure of polymers [29]. With the increase of aging time, the concentration and depth of traps in the sample increased, owing to the polar groups and double bond structures introduced by the cracking and oxidation of molecular chains. The directional movement of carriers in the high field would be hindered by more charges captured in deep traps. This has a positive effect on short-time breakdown field strength [30]. While, the weak areas formed by cracking, oxidation and cross-linking would lead to the concentration of local electric field which reduced the breakdown strength of insulation [31]. As the aging time prolonged, the structure and arrangement of polymer chains changed. The effect of aging on space charge distribution and carrier motion in electric field was different. Finally, the short-term breakdown strength decreased slightly during the aging process.

### 3.4. Tensile Testing

**Figure 6:**
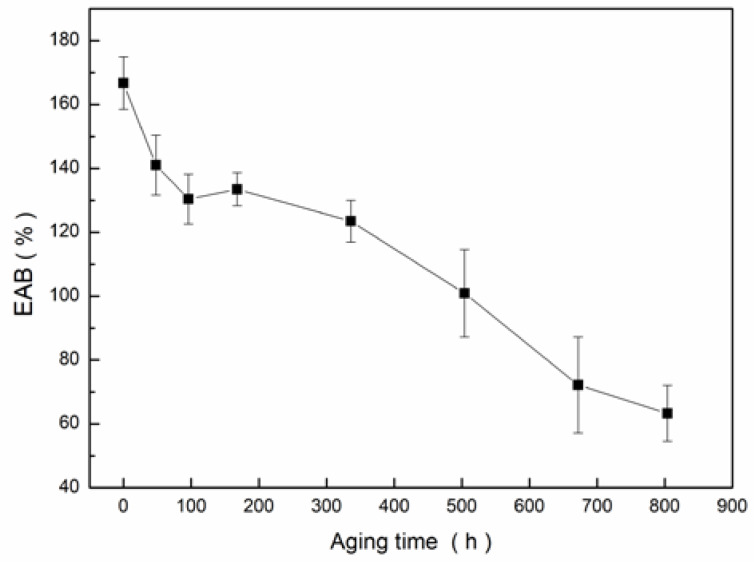
Elongation at break versus time of thermal-oxidative aging at 165 °C.

The tensile test curve of samples at different aging stages is shown in Figure 6. With the increase of aging time, the EAB decreased continuously. At the early stage of aging, the EAB decreased obviously, and after 672 h of aging, the EAB decreased to less than half of the unaged sample, which indicated that the insulation reached the end of its life [32].

At the case that the end of life was characterized by EAB, the real permittivity of insulation decreased from 5.02 to 4.88 and tanδ decreased by an order of magnitude at an operating frequency of 50 Hz. The DC insulation resistivity increased 38 times, and the AC breakdown field strength was slightly changed. It could be seen that the change of resistivity and loss was obvious during the aging process.

## 4. Conclusions

A new kind of cable insulation for NPPs was evaluated for thermal-oxidative degradation. The samples were aged at 165 °C from 48 h to 804 h, and the thermal, chemical, dielectric and tensile characteristics were compared during aging. 

FTIR results showed that the aging products such as hydroxyl, carboxylic acid, alcoholic and ester groups were produced and a large number of double bonds were also formed during thermal-oxidative aging. The active groups produced by aging promoted the process of oxidative induction, which showed that the initial temperature and peak temperature of oxidative induction decreased.

Aging had a great influence on the conductivity of the samples, which showed that the volumetric resistivity of the samples increased obviously during aging. After aging for 804 h, the volumetric resistivity of the sample was about 62 times of that of the unaged sample, and the low frequency conductivity loss was reduced. Aging had little effect on AC breakdown.

When the EAB decreased to half of the initial value, the insulation had undergone 672 h of aging. At this time, at 50 Hz operating frequency, tanδ decreased by an order of magnitude, and DC insulation resistivity increased 38 times. It can be seen that, for this sample, insulation resistivity and loss were expected to become the characteristic parameters of aging assessment.
